# The use of proteomics for blood biomarker research in premature infants: a scoping review

**DOI:** 10.1186/s12014-021-09316-y

**Published:** 2021-04-14

**Authors:** Natasha Letunica, Tengyi Cai, Jeanie L. Y. Cheong, Lex W. Doyle, Paul Monagle, Vera Ignjatovic

**Affiliations:** 1 Haematology Research Laboratory, Murdoch Children’s Research Institute, Parkville, Australia; 2 Victorian Infant Brain Study (VIBeS), Murdoch Children’s Research Institute, Parkville, Australia; 3 Department of Paediatrics, The University of Melbourne, 50 Flemington Road, Parkville, Australia; 4 Department of Clinical Haematology, Royal Children’s Hospital, Parkville, Australia; 5 Department of Obstetrics and Gynecology, The Royal Women’s Hospital, Parkville, Australia; 6 Newborn Research, The Royal Women’s Hospital, Parkville, Australia

**Keywords:** Proteomics, Biomarkers, Premature Infants, Prematurity

## Abstract

Over the last decade, the use of proteomics in the setting of prematurity has increased and has enabled researchers to successfully identify biomarkers for an array of associated morbidities. The objective of this scoping review was to identify the existing literature, as well as any knowledge gaps related to proteomic biomarker discoveries in the setting of prematurity. A scoping review was conducted using PubMed, Embase and Medline databases following the Preferred Reporting Items for Systematic reviews and Meta-Analyses extension for Scoping Reviews (PRISMA-ScR) guidelines. The study selection process yielded a total of 700 records, of which 13 studies were included in this review. Most studies used a tandem Mass Spectrometry (MS/MS) proteomics approach to identify key biomarkers. The corresponding studies identified proteins associated with retinopathy of prematurity (ROP), bronchopulmonary dysplasia (BPD), necrotising enterocolitis (NEC), late onset sepsis (LOS) and gestational age. This scoping review demonstrates the limited use of proteomics to identify biomarkers associated with severe complications of prematurity. Further research is warranted to identify biomarkers of other important morbidities associated with prematurity, such as intraventricular haemorrhage (IVH) and cerebral palsy, and to investigate the mechanisms associated with these outcomes.

## Introduction

Proteomics is a methodological approach that allows for the analysis of many proteins simultaneously and has been successful in identifying many novel disease biomarkers [1]. Proteomic methodologies have been previously used in varying contexts, such as discovering biomarkers of diabetic nephropathy and identifying novel diagnostic markers of cancer [2, 3]. Plasma proteomics is advantageous as it only uses a small volume of blood to study hundreds and sometimes thousands of proteins, and can identify changes in protein expression that may occur with age and disease [4]. Proteomics is not limited to analysis of blood samples, and enables the use of biological fluids such as saliva and urine, and tissue samples (e.g. tumours) [5]. Due to the small volume required for analysis, plasma proteomics has become increasingly popular and has enabled investigations of plasma proteins in vulnerable populations such as in paediatrics, as well as in critically ill patients, where blood may be scarce and not readily available for research purposes [4].

Preterm birth is the leading cause of death among the paediatric population globally [6]. With major technological advances in neonatal care over the last few decades, there has been an increase in survival of infants born preterm (< 37 weeks’ gestation), in particular those born extremely preterm (< 28 weeks’ gestation) [7]. Despite the technological advances that have improved survival in these vulnerable populations, preterm birth is associated with significant morbidities including intraventricular haemorrhage (IVH), necrotising enterocolitis (NEC), bronchopulmonary dysplasia (BPD), and neurosensory impairments [8].

Within the last decade proteomics has enabled researchers to identify predictive biomarkers of NEC in preterm infants using buccal swabs [9]. More specifically, plasma proteomics has previously identified proteins that may play a role in the development of retinopathy of prematurity [10]. However, to date there has been limited research into plasma protein biomarkers in predicting other outcomes in preterm infants. Consequently, a scoping review was conducted to understand the current state of knowledge in this space, and to identify knowledge gaps that could be addressed by future studies. A preliminary search of MEDLINE, PubMed, JBI Evidence Synthesis and Embase was conducted and did not identify any current systematic reviews or scoping reviews on this topic. Thus, this review is novel and will make a significant contribution to the understanding and knowledge in the use of proteomics in preterm infants.

## Review question

The following research question was formulated using the PCC (Population, Concept, Context) framework: *What is the existing proteomic evidence of blood biomarker research in the setting of prematurity?*

## Methods

### Study design

This scoping review was conducted based on the Preferred Reporting Items for Systematic reviews and Meta-Analyses extension for Scoping Reviews (PRISMA-ScR) Checklist [11].

### Search strategy

The following three electronic databases: Medline, Embase and PubMed were searched on the 24th September 2020 for all peer-reviewed studies. An additional search for grey literature was conducted using the OpenGrey and GreyLit databases. The specific search terms used for each database are detailed in Appendix A. In summary, studies included in this review were identified using the search terms [‘preterm’ OR ‘premature’) AND [‘proteome’ OR ‘protein-analysis’] AND [‘blood-protein’ OR [biomarker’], as well as including derivatives of these terms. Studies identified in this review were limited to those written in the English language and conducted in humans only. Studies retrieved using these search terms and parameters were screened by two authors (NL and TC), initially focusing on the eligibility of the studies’ titles and abstracts using the following inclusion and exclusion criteria.

### Selection criteria

Inclusion criteria: (I) infants born preterm (< 37 weeks), (II) blood proteome assessed, (III) primary research, (IV) English language and (V) human study.

Exclusion criteria: (I) infants born at term or post-term (≥ 37 weeks), (II) proteome of other biological samples (e.g. saliva or urine) assessed, (III) case report, review, conference abstract or editorial correspondence and (IV) animal studies.

### Data extraction and charting

Studies that were chosen for full-text assessment were assessed by NL and TC and with any discrepancies and uncertainties, a third reviewer (VI) was to assess the studies. Data extracted included publication year, disease/outcome assessed, aims, study population, comparative groups, proteomic methodology, protein-pathway analysis, key findings and study limitations. The detailed assessment for each critically reviewed study is presented in Table 1.

**Table 1:** Summary of included studies in the scoping review of proteomics in setting of prematurity

Author Year Country	Outcome	Aim	Population	Comparative groups (n =)	Proteomic methods	Pathway analysis	Key findings	Limitations
Byung et al. [23] 2004 Korea	PDA	To investigate the usefulness of rapid BNP assay as a diagnostic marker of symptomatic PDA in preterm infants	Preterm infants aged 25–34 weeks’ gestation	Symptomatic PDA (n = 23) Control (n = 43)	Immunoassay kits	None	Circulating BNP measurements correlated with clinical and echocardiographic assessments of PDA BNP concentration was significantly higher in the infants with symptomatic PDA 3 days after birth BMP concentration measurements were correlated with ductal shunts	Not listed
Ng et al. [16] 2010 Hong Kong	LOS NEC	To identify novel biomarkers for early and accurate diagnosis of NEC and/or septicaemia in premature infants Develop a novel clinical strategy of antibiotic treatment in different risk categories of infants	Infants born < 31 weeks’ gestation and with a birth weight of < 1500 g	Sepsis/NEC (n = 77) No sepsis infants (n = 77)	MALDI-TOF MS Immunoassay kits Protein microarray 2D-Gel Electrophoresis	None	The ApoSAA score can potentially formulate antibiotic treatment strategies for suspected LOS and NEC patients The ApoSAA Score equation is practical and clinically useful for accurate identification of NEC and LOS in preterm infants Proteins that are useful biomarkers of NEC and LOS: Pro-apoC2 and a des-arginine variant of SAA	Proteomic protocol may not differentially detect low-plasma concentration proteins
Stewart et al. [12] 2015 UK	LOS NEC	To investigate serum and metabolome longitudinally in preterm infants with NEC and LOS	Infants born 23–30 weeks’ gestation	NEC (n = 6) LOS (n = 4) Control (n = 9)	LC–MS/MS	None	All proteins and metabolites were comparable among all patient groups C-reactive protein increased in all NEC patients Upregulated proteins associated with NEC diagnosis: C-reactive protein (1–205), MIF and SAA-2 Proteins associated with LOS diagnosis: Haptoglobin, transthyretin and U5 small nuclear ribonucleoprotein	Study was not sufficiently powered to determine biomarkers for clinical diagnosis Serum samples were salvaged post routine clinical tests
Ruiz-Gonzalez et al. [17] 2015 Spain	IUGR	To analyse and identify serum proteome changes in IUGR and AGA infants	Infants born 29- ≥ 37 weeks’ gestation	Very preterm (29–32 weeks’ gestation) (n = 28) Moderate preterm (33–36 weeks’ gestation) (n = 30) Term (≥ 37 weeks’ gestation) (n = 30)	MALDI-TOF MS 2D-Gel Electrophoresis Western blot	None	MBOAT7 was only detected in IUGR across all GA groups Lower levels of APOL1 and SUMO3 were detected in UGR compared to AGA FCN2 was downregulated in IUGR after one week in the very preterm group, whereas TF was upregulated in the very preterm and term groups	Extremely preterm infants (< 29 weeks) were not included in the study
Lynch et al. [18] 2016 USA	ROP	Identify plasma proteins associated with ROP	Infants born < 31 weeks’ gestation or birth weight < 1500 g	No ROP (n = 23) Clinically significant ROP (n = 12) Low-grade ROP (n = 27)	SOMAscan proteomic assay	None	Proteins associated with clinically significant ROP: MnSOD, CRDL1 and PCSK9 MnSOD could be used as a therapeutic intervention target Proteins associated with a high risk of ROP included: FGF-19, MST1R, LH, cystatin M and Plasminogen IGFBP-7 was linked to the signalling pathway for ROP	Small sample size Proteomic analysis was conducted on one sample from neonatal period
Suski et al. [13] 2018 Poland	GA	To compare plasma proteome compositions in preterm infants from varying gestational ages To identify signalling pathways that could be differentially regulated due to the duration of a pregnancy	Infants born < 30 weeks’ gestation	Preterm Group 1 (< 26 weeks’ gestation) (n = 19) Preterm Group 2 (27–28 weeks’ gestation) (n = 19) Preterm Group 3 (29–30 weeks’ gestation) (n = 19)	iTRAQ LC–MS/MS	None	Protein changes between gestation ages across several pathways for inflammation, immunomodulation, complement activation and coagulation As gestational age increased there was an increase in plasma protease inhibitor (C1Inh) and fibrinogen isoforms As gestational age increased there was a decrease in Complement C3, Factor V and C4-A Concentration of LRG1 increased over time SAP correlated with gestation age Significant changes in plasma concentrations of Apolipoprotein compositions, specifically Apo-D	Not listed
Suski et al. [14] 2018 Poland	Signalling Pathways	To analyse plasma proteome changes in preterm infants that are stratified by their gestational age in order to identify proteins of malfunctioning signalling pathways	Infants born < 30 weeks’ gestation	Preterm Group 1 (< 26 weeks’ gestation) (n = 19) Preterm Group 2 (27–28 weeks’ gestation) (n = 19) Preterm Group 3 (29–30 weeks’ gestation) (n = 19)	iTRAQ LC–MS/MS	None	Changes in plasma protein concentrations were associated with preterm delivery LRG1 was negatively correlated with gestation age Downregulation of ORM 1 and 2 isoforms ZAG and afamin downregulated in all groups Changes in the inflammatory, coagulation and complement pathways identified among infants born preterm	Not listed
Wagner et al.[21] 2018 USA	PVD	Identify proteins associated with pathogenesis of PVD	Preterm infants aged 23–29 weeks’ gestation	PVD (n = 44) Non-PVD group (n = 56)	SOMAscan proteomic assay	None	18 proteins associated with PVD at day 7 (PF-4, MST1R, APP and STK16) Proteins associated with novel pathways: Platelet degranulation, signalling by MST1	Single centre study Circulating proteins may not correctly represent target organ
Zasada et al. [10] 2018 Poland	ROP	To identify biomarkers of ROP To validate the findings with a gene expression study	Infants born < 30 weeks’ gestation	Preterm infants with ROP (n = 28) Preterm infants without ROP (n = 29)	iTRAQ Protein Microarray MS/MS	None	Significant difference in 33 proteins among those who developed ROP compared with infants who did not Concentrations of complement C3 and fibrinogen increased in infants who developed ROP Microarray results for fibrinogen did not validate the findings from the proteomic analysis	Results may not be generalised due to differences across varying NICUs An additional validation method could have been used to strengthen the reported findings
Zasada et al. [15] 2019 Poland	BPD	To identify plasma biomarkers of BPD and provide a further molecular understanding of BPD	Infants born < 30 weeks’ gestation	Preterm infants with BPD (n = 36) Preterm infants without BPD (n = 21)	iTRAQ MS/MS	None	Infants with BPD had a decrease in the following protein concentrations: afamin, gelsolin, apolipoprotein A-1 and galectin-3 binding protein t 36 weeks’ postmenstrual (PMA) infants with BPD had increasing plasma concentrations of TF	Sample size of infants with severe BPD is small An additional validation method could have been used to strengthen the reported findings
Arjaans et al. [19] 2020 USA	BPD PH	Determine changes in circulating angiogenic peptides during the first week of life and their association with developing BPD and PH later in life Determine peptides and relevant signalling pathways associated with risk of BPD and PH	Infants born < 34 weeks’ gestation and a birthweight between 500 and 1250 g	No BPD (n = 20) Mild BPD (n = 34) Moderate BPD (n = 26) Severe BPD (n = 22)	SOMAscan proteomic assay	Reactome	Proteins associated with BPD severity include: FGF-19, PF-4, CTAP-III and PDGF-AA Proteins associated with BPD diagnosis: PF-4, VEGF121, ANG-1, ANG-2, BMP10 AND HGF Increasing BMP10 levels were associated with Preterm infants developing BPD and PH later in life	Relatively small sample size Circulating proteins may not represent expression in lung tissue
Tosson et al. [24] 2020 Egypt	Sepsis	To investigate S100A12 and additional cytokines as biomarkers for neonatal sepsis	Infants born 24–36 weeks’ gestation	Controls (n = 22) Not infected (n = 22) Infection probable (n = 37) Infected (n = 37)	ELLSA Magnetic bead array assay	None	S100A12 demonstrated high specificity and sensitivity between infected and control groups IL-6 and IL-10 were significantly different between infected and control group S100A12 was also significantly different among control and infected groups	Not listed
Zhong et al. [25] 2020 Sweden	Blood protein profiles	To investigate protein profiles in extremely preterm infants	Infants born < 28 weeks’ gestation	Extremely preterm infants (n = 14)	Multiplex PEA technology	None	Proteins that increased after birth: C3dCR2, Factor VII, Factor XI, INHBC, SELL, IL2-RA and GP6 Proteins that decreased after birth: COLEC12, IGFBP-1, FSTL3, GDF15 and CGA Infants born extremely preterm have similar serum profiles directly at birth which changes dramatically during the first week of life	Small sample size Some infants received blood products during the study period, which could have impacted the results

ROP: retinopathy of prematurity; PVD: pulmonary vascular disease; PH: pulmonary hypertension; LOS: late onset sepsis; BPD: bronchopulmonary dysplasia; NEC: necrotising enterocolitis; GA: gestational age;Pro-apoC2: Proapolipoprotein CII; SAA: serum amyloid A; MALDI-TOF MS: matrix assisted laser desorption ionization-time of flight mass spectrometry; MnSOD: mitochondrial superoxide dismutase; CRDL1: chordin-like protein 1;PCSK9: proprotein convertase subtilisin/kexin type 9; FGF-19: Fibroblast growth factor 19; MSP: hepatocyte growth factor-like protein; LH: luteinizing hormone; IGFBP-7: insulin-like growth factor-binding protein-7; iTRAQ: isobaric tags for relative and absolute quantitation; LC–MS/MS: liquid chromatography and tandem mass spectrometry; C1Inh: C1-inhibitor; LRG1: leucine-rich alpha-2-gylcoprotein; SAP: serum amyloid P-complement; Apo-D: apolipoprotein D; ZAG: zinc-alpha-2-glycoprotein; ORM: Orosomucoid; MST1: macrophage stimulating 1; PF-4: platelet factor 4; MSP: macrophage-stimulating receptor protein; APP: amyloid precursor protein; STK16: serine/threonine-protein kinase 16; CTAP-III: connective tissue-activating peptide III; PDGF-AA: Platelet-derived growth factor AA; VEGF121: Vascular endothelial growth factor 121; ANG-1: Angiopoietin 1; ANG-2: Angiopoietin 2; BMP10: Bone morphogenetic protein 10; HGF: Hepatocyte growth factor; PEA: proximity extension assays; C3dCR2: complement C3d Receptor 2; COLEC12: collectin subfamily member 12; INHBC: inhibin beta C subunit; SELL: selectin L; IL2-RA: interleukin 2 Receptor alpha; GP6: glycoprotein 6 platelet; IGFBP-1: insulin-like growth factor-binding protein-1; FSTL3: follistatin like 3; GDF15: growth differentiation factor 15; CGA: glycoprotein hormone alpha polypeptide; ELLSA: enzyme-linked immunosorbent assay; MIF: macrophage migration inhibitory factor; IUGR: Intrauterine growth restriction; AGA: adequate gestational age; MBOAT7: lysophospholipid acyltransferase 7; SUMO3: small ubiquitin-related modifier 3; FCN2: ficolin-2; TF: serotransferrin; PDA: patent ductus arteriosus; BNP: B-type natriuretic peptide

## Results

**Fig. 1:**
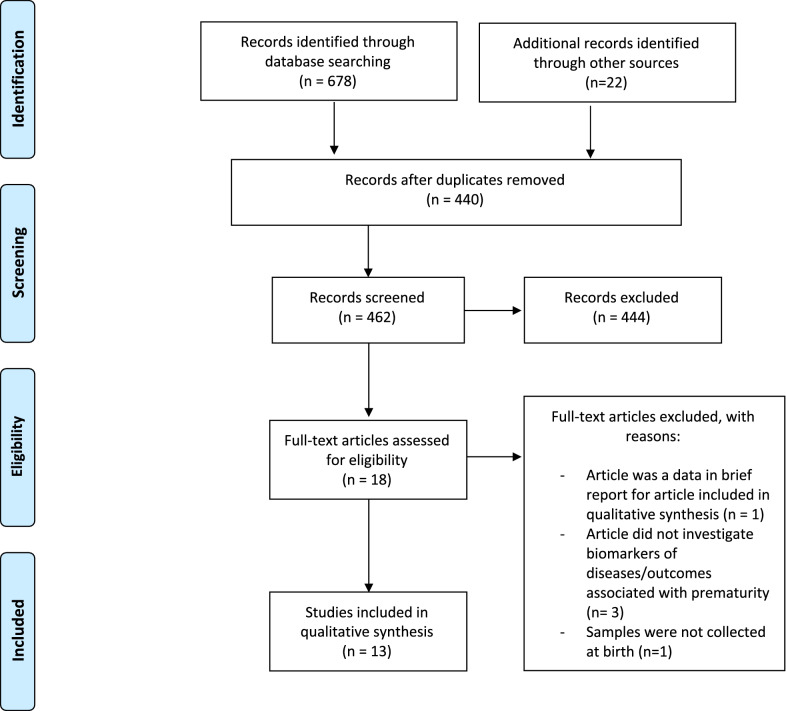
Summary of the study selection process for the scoping review

**Fig. 2:**
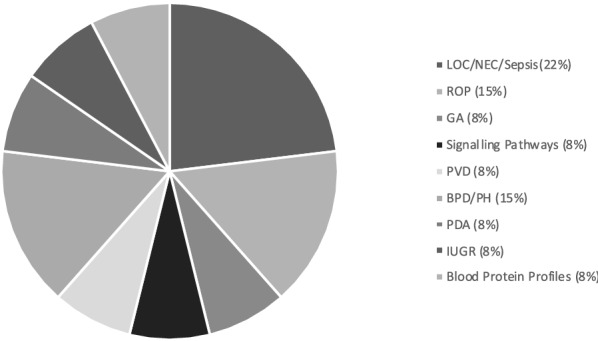
Blood proteomic studies identified were primarily conducted in the setting of LOC/NEC (23%, 3 studies) and ROP (15%, 2 studies)

The initial search identified 678 studies using the scoping review search strategy, with an additional 22 studies identified using the grey literature search. After the removal of duplicates, 462 publications remained for title and abstract screening. A vast majority of studies (n = 444, 96%) were excluded due to not fulfilling the inclusion criteria or having no relevance to the topic of prematurity and blood biomarker discoveries. Eighteen studies underwent full-text review, with three studies excluded because they did not primarily investigate biomarkers of disease and outcomes. One study of children born preterm did not collect samples at birth and one study presented data in brief report, which did not include any proteomic data. Figure 1 illustrates the article screening and selection process, following the PRISMA guidelines (Fig. 2).

### Description of included studies

**Fig. 3:**
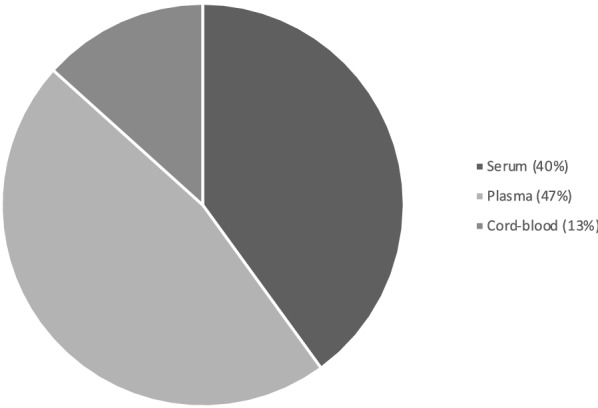
Sample types used in the identified studies were primarily conducted using plasma (47%, 7 studies) and serum (40%, 6 studies)

A total of thirteen studies met the inclusion criteria for this scoping review and are summarised in Table 1. Eleven of the thirteen included studies investigated proteins and their associations with known outcomes of prematurity. The participant gestational age at birth ranged from < 23 to 37 weeks, with sample sizes varying from 4 to 77 participants. Most studies used a tandem Mass Spectrometry method (MS/MS) to analyse the proteins of interest [10, 12–15]. Three of the fourteen studies also conducted protein validation and completed this task using protein microarray and immunoassay techniques [10, 16, 17]. Approximately half of the studies (n = 7, 47%) were completed using plasma samples (Fig. 3). The proteins identified as proteins of interest across the 13 studies included in this scoping review, with reference to the specific study/ies are summarised in Table 2.

**Table 2:** Proteins identified in the studies included in this scoping review

Protein	Gene	UniProt accession number	Molecular function	Biological process	Study
Natriuretic peptides B	NPPB	P16860	Diuretic hormone activity/ hormone receptor binding	Cell surface receptor signalling pathway/ body fluid secretion	[23]
Apolipoprotein C-II	APOC2	P02655	Lipoprotein lipase activator activity/ lipid binding	High-density lipoprotein particle remodelling/ retinoid metabolic process	[16]
Serum Amyloid A	SAA	P0DJI8	G protein-coupled receptor/heparin binding	Activation of MAPK activity/ acute-phase response	[12, 16]
C-reactive protein (1–205)	CRP	P02741	Calcium ion/ choline binding	Complement activation/ innate immune response	[12]
Macrophage migration inhibitory factor	MIF	P14174	Cytokine activity/ receptor binding	Innate immune response/inflammatory response	[12]
Serum amyloid A-2	SAA-2	P0DJI9	G protein-coupled receptor/heparin binding	Acute-phase response	[12]
Transthyretin	TTR	P02766	Hormone activity	Cellular protein metabolic process/ extracellular matrix organization	[12]
Haptoglobin	HP	P00738	Antioxidant activity/ haemoglobin binding	Acute inflammatory response	[12]
U5 small nuclear ribonucleoprotein	SNRNP40	Q96DI7	RNA binding	RNA splicing and processing	[12]
Lysophospholipid acyltransferase 7	MBOAT7	Q96N66	Lysophospholipid acyltransferase activity	Lipid modification/regulation of triglyceride metabolic process	[17]
Apolipoprotein L1	APOL1	O14791	Chloride channel activity/ lipid binding	Cellular Protein Metabolic Process/ cholesterol metabolic process	[15, 17]
Small ubiquitin-related modifier 3	SUMO3	P55854	Protein tag/ ubiquitin-like protein ligase binding	Negative regulation of DNA binding	[17]
Ficolin-2	FCN2	Q15485	Antigen/Calcium-dependant protein binding	Complement activation	[17]
Serotransferrin	TF	P02787	ferric iron binding	Cellular iron ion homeostasis	[15, 17]
Mitochondrial superoxide2	SOD2	P04179	DNA/enzyme binding	Cellular response to oxidative stress	[18]
Chordin-like protein 1	CHRDL1	Q9BU40	Developmental protein	BMP signalling pathway/ post-translational protein modification	[18]
Proprotein convertase subtilisin/kexin type 9	PCSK9	Q8NBP7	Apolipoprotein binding	Apoptotic process	[18]
Fibroblast growth factor 19	FGF-19	O95750	Fibroblast Growth Factor Receptor Binding	MAPK cascade/ positive regulation of protein phosphorylation	[18, 19]
Macrophage-stimulating protein receptor	MST1R	Q04912	ATP/ enzyme binding	Cell migration/ hepatocyte growth factor receptor signalling pathway	[18, 21]
Glycoprotein hormones alpha chain	CGA	P01215	Follicle-stimulating hormone activity	Peptide hormone processing	[25]
Cystatin-M	CST6	Q15828	Cysteine-Type Endopeptidase Inhibitor Activity	Anatomical structure morphogenesis	[18]
Plasminogen	PLG	P00747	Apolipoprotein Binding/ protein domain specific binding	Blood coagulation/ cellular protein metabolic process	[18]
Insulin-like growth factor-binding protein 7	IGFBP-7	Q16270	Insulin-Like Growth Factor Binding	Cell Adhesion/ cellular protein metabolic process	[18]
Plasma protease C1 inhibitor	SERPING1	P05155	Serine-Type Endopeptidase Inhibitor Activity	Blood coagulation, intrinsic pathway/ complement activation, classical pathway	[13]
Complement C3	C3	P01024	C5L2 anaphylatoxin chemotactic receptor binding	Cellular protein metabolic process	[10, 13]
Coagulation factor V	F5	P12259	Copper ion binding	Cellular protein metabolic process/ platelet degranulation	[13]
Complement C4-A	C4A	P0C0L4	Endopeptidase inhibitor activity	Cellular protein metabolic process/ regulation of complement activation	[13]
Leucine-rich alpha-2-glycoprotein	LRG1	P02750	Transforming growth factor beta receptor binding	Neutrophil degranulation	[13, 14]
Serum amyloid P-component	APCS	P02743	Calcium ion/ carbohydrate binding	cellular protein metabolic process/ complement activation	[13]
Apolipoprotein D	APOD	P05090	Cholesterol binding/ lipid transporter activity	Angiogenesis/ lipid metabolic process	[13]
Alpha-1-acid glycoprotein 1	ORM 1	P02763	Inflammatory response	Platelet/ neutrophil degranulation	[14]
Zinc-alpha-2-glycoprotein	AZGP1	P25311	Protein transmembrane transporter activity	Transmembrane transport/ retina homeostasis	[14]
Platelet factor 4	PF4	P02776	Chemokine activity/ heparin binding	G protein-coupled receptor signalling pathway	[19, 21]
Amyloid-beta precursor protein	APP	P05067	Acetylcholine receptor binding	Cellular protein metabolic process	[21]
Serine/threonine-protein kinase 16	STK16	O75716	ATP binding/ protein serine/threonine kinase activity	Protein autophosphorylation	[21]
Afamin	AFM	P43652	Fatty acid/ vitamin E binding	Vitamin transport/ protein stabilisation	[14, 15]
Gelsolin	GSN	P06396	Actin/ calcium ion binding	Cellular protein metabolic process	[15]
Galectin-3	LGALS3	P17931	Oligosaccharide/ RNA binding	Neutrophil degranulation/ innate immune response	[15]
Vascular endothelial growth factor A	VEGFA	P15692	Vascular endothelial growth factor receptor binding	Activation of protein kinase activity/ angiogenesis	[19]
Angiopoietin-2	ANGPT2	O15123	Metal ion binding/ receptor tyrosine kinase binding	Angiogenesis/ leukocyte migration	[19]
Angiopoietin-1	ANGPT1	Q15389	Receptor tyrosine kinase binding	Angiogenesis/ leukocyte migration	[19]
Bone morphogenetic protein 10	BMP10	O95393	Growth factor/ cytokine activity	Cell adhesion/ BMP signalling	[19]
Hepatocyte growth factor receptor	MET	P08581	ATP binding/ protein tyrosine kinase activity	cell surface receptor signalling pathway/ cell migration	[19]
Protein S100-A12	S100A12	P80511	Calcium/ion binding	Cytokine secretion/ inflammatory response	[24]
Interleukin-6	IL6	P05231	Cytokine/ growth factor activity	Cellular protein metabolic process/ acute-phase response	[24]
Interleukin-10	IL10	P22301	Cytokine/ growth factor activity	B cell differentiation/ cytokine-mediated signalling pathway	[24]
Complement receptor type 2	CR2	P20023	Complement binding/ DNA binding	B cell differentiation/ immune response	[25]
Coagulation factor VII	F7	P08709	Calcium ion binding/ signalling receptor binding	Blood coagulation-extrinsic pathway	[25]
Coagulation factor XI	F11	P03951	Heparin binding	Blood coagulation-intrinsic pathway/ plasminogen activation	[25]
L-selectin	SELL	P14151	Calcium ion binding	Leukocyte migration/ regulation of immune response	[25]
Interleukin-2 receptor subunit alpha	IL2RA	P01589	Interleukin-2 binding/ receptor activity	cytokine-mediated signalling pathway	[25]
Platelet glycoprotein VI	GP6	Q9HCN6	Collagen binding/ signalling receptor activity	Blood coagulation/ platelet activation/ leukocyte migration	[25]
Collectin-12	COLEC12	Q5KU26	Galactose binding/ low-density lipoprotein particle binding	Receptor-mediated endocytosis/ regulation of immune response	[25]
Follistatin-related protein 3	FSTL3	O95633	Activin/ fibronectin binding	Cellular protein metabolic process/ cell differentiation	[25]
Growth/differentiation factor 15	GDF15	Q99988	BMP receptor binding/ growth factor activity	Activation of MAPK activity/ BMP signalling	[25]
Insulin-like growth factor-binding protein 1	IGFBP1	P08833	Insulin-like growth factor binding	Cellular protein metabolic process	[25]

### Retinopathy of prematurity (ROP)

Two studies investigated the outcome associated with prematurity, ROP [10, 18]. ROP is seen most commonly among infants born very preterm (< 32 weeks’ gestational age) or < 1250 g birth weight. Abnormal blood vessel development occurs in the retina in response to oxygen exposure, which can lead to retinal detachment and blindness in severe cases [18]. Currently there is no existing method to predict the occurrence of ROP in infants born preterm or born with a low birth weight and all high-risk infants are routinely screened. Hence, a proteomic approach was adopted to identify underlying biomarkers of the disease [10, 18]. Several biomarkers of the complement and inflammatory system were identified in infants who developed ROP [10]. Lynch et al. identified mitochondrial Superoxide dismutase (MnSOD), an antioxidant located in the mitochondria, as a potential therapeutic target for significant ROP [18].

### Bronchopulmonary dysplasia (BPD) and pulmonary vascular disease (PVD)

Two of the thirteen included studies investigated plasma proteins and their association with BPD [15, 19]. BPD is a chronic lung disease that affects infants born preterm [20]. Arjaans et al. implemented the use of a SOMAscan proteomic assay, whereas Zasada et al. utilised MS/MS approach to identify key biomarkers of BPD. Both studies identified several proteins that may be used in future diagnosis of BPD as well associations between severity and disease prognosis [15, 19]. Wagner et al. investigated plasma proteins and their association with the pathogenesis of PVD, a term used to describe abnormal function and vascular growth of the lungs. They identified 18 proteins that were associated with PVD, including proteins associated with growth factors, angiogenesis and the extracellular matrix [21]. The protein analysis conducted by Wanger et al. also identified proteins of several different biological process pathways (e.g. Tissue Inhibitor of Metalloproteinases 3 (TIMP-3) used in platelet degradation and Bone proteoglycan II, involved in degradation of the extracellular matrix (ECM)) that may be associated with PVD.

### Necrotising enterocolitis (NEC) and late onset sepsis (LOS)

Two of the thirteen studies examined biomarkers for NEC and LOS [12, 16]. Ng et al. investigated biomarkers for the early diagnosis of NEC among preterm infants. Ng et al. investigated their samples with a variety of proteomic methods, which included matrix-assisted laser desorption/ionisation (MALDI-ToF), 2D Gel-Electrophoresis (2DGE). The results of the discovery component of the study were validated using commercially available immunoassay kits and protein microarrays. Ng et al. identified a des-arginine variant of serum amyloid A (SAA) and Proapolipoprotein CII (Pro-apoC2) as very promising biomarkers of late-onset septicaemia and NEC [16]. Stewart et al. investigated the serum and metabolome of preterm infants with NEC and LOS longitudinally with a LC- MS/MS technique. Among all patient groups investigated the proteins and metabolite were comparable, with 12 proteins (e.g. Serum Amyloid A-2 and Haptoglobin) associated with NEC and LOS diagnosis [12]. Interestingly, the only protein common across the two studies was SAA [12, 16].

### Gestational age and signalling pathways

Suski et al. completed several studies [13, 14] investigating plasma proteome changes in preterm infants comparing gestational ages [13] and malfunctioning proteins in various signalling pathways [14]. Utilising a tandem MS approach they were able to identify proteomic changes across varying gestational ages for several pathways which include; coagulation, inflammation, complement activations and immunomodulation [13, 14]. Suski et al. also observed Complement C3, Factor V and Complement C4-A were associated with gestational age [13]. LRG1 was the only common protein identified across the two studies [13, 14].

## Discussion

In this scoping review we identified 13 primary studies that used proteomics to identify blood protein biomarkers in the setting of prematurity that used either plasma or serum as the sample which was analysed. It is important to note that studies conducted in serum cannot be directly compared to studies conducted in plasma as these are two entirely different samples. Unlike plasma which is prepared only via centrifugation, Preparation of serum entails formation and removal of a blood clot activating not only coagulation proteins but also changing the concentration of inflammatory proteins, a scenario that reflects the manipulation itself and not the physiological setting. Similarly, a cord-blood sample is different to the blood sample collected from babies at birth, due to differences in the vasculature of the umbilical cord and blood vessels within the newborn. Our findings indicate that the focus of research in the setting of blood protein biomarkers in the setting of prematurity focused on several diseases, such as ROP, BPD, LOS and NEC. However, there has been a lack of research focusing into other outcomes known to be associated with preterm birth such as cerebral palsy, intraventricular haemorrhage, or hypertension. To our best knowledge, none of the findings from the studies included in our scoping review have been translated into the clinical setting. Blood proteomic studies within this population may reflect a lack of collaboration between clinicians and proteomic experts, as well as difficulty in accessing samples from premature babies, factors that could be overcome, particularly in research institutes associated with tertiary hospitals [22].

### Limitations of current published studies

The main limitation of the studies included in this review are the small sample sizes represented in those studies. Future studies should be adequately powered, and a shift of the primary focus from not only understanding mechanism of disease, but also on  identifying proteins that are associated with outcomes or disease and which can be used in the clinical setting to improve outcomes for premature infants.

### Conclusions

This scoping review  identified a paucity of evidence around biomarker discoveries in the population of preterm infants. Several proteomic methods, including tandem mass spectrometry, immunoassays, and MALDI-TOF MS, have been used to identify biomarkers for various outcomes (e.g. ROP and BPD) associated with preterm birth. This review identifies the need for future research focusing on biomarkers to understand the possible mechanisms related to preterm birth, as well as to identify predictive protein biomarkers for complications or long-term sequelae associated with preterm birth, such as intraventricular haemorrhage and hypertension.

## Data Availability

Not applicable.

## A. 1. PubMed database

1. “Preterm” OR “pre-term” OR “prematur*”
2. “Proteom*” OR “protein-analysis”
3. “Blood-protein*” OR “serum-protein*” OR “plasma-protein*” OR “biomarker*” OR “marker*”
4. 1 and 2 and 3
5. (“Animal” OR “animals” OR “rat” OR “rats” OR “mouse” OR “mice” OR “swine” OR “porcine” OR “murine” OR “sheep” OR “lamb” OR “lambs” OR “pig” OR “pigs” OR “piglet” OR “piglets” OR “rabbit” OR “rabbits” OR “cat” OR “cats” OR “dog” OR “dogs” OR “cattle” OR “bovine” OR “monkey” OR “monkeys” OR “trout” OR “marmoset” OR “marmosets”) NOT (“human” OR “humans” OR “patient” OR “patients” OR “newborn*” OR “baby” OR “babies” OR “neonat*” OR “infan*” OR “toddler*” OR “pre-schooler*” OR “preschooler*” OR “kindergarten” OR “boy” OR “boys” OR “girl” OR “girls” OR “child” OR “children” OR “childhood” OR “adolescen*” OR “pediatric*” OR “paediatric*” OR “youth*” OR “teen” OR “teens” OR “teenage*” OR “school-aged*” OR “school-child*” OR “school-girl*” OR “school-boy*” OR “schoolgirl*” OR “schoolboy*” OR “man” OR “men” OR “woman” OR “women” OR “adult” OR “adults” OR “middle-age*” OR “elderly”)
6. 5 not 6
7. Limit to English language

## A. 2. Embase database

1. Prematurity/
2. Exp low birth weight/
3. (Preterm or pre-term or prematur*).mp.
4. 1 or 2 or 3
5. Exp proteomics/
6. Proteome/
7. Exp *protein analysis/
8. Proteom*.tw,kw,dq.
9. 5 or 6 or 7 or 8
10. Exp plasma protein/
11. (Blood-protein* or serum-protein* or plasma-protein*).tw,kw,dq.
12. Biological marker/
13. (Biomarker* or marker*).tw,kw,dq.
14. 10 or 11 or 12 or 13
15. 4 and 9 and 14
16. (Rat or rats or mouse or mice or swine or porcine or murine or sheep or lamb or lambs or pig or pigs or piglet or piglets or rabbit or rabbits or cat or cats or dog or dogs or cattle or bovine or monkey or monkeys or trout or marmoset or marmosets).ti. and animal experiment
17. Animal experiment/ not (human experiment/ or human/)
18. Case report/
19. 15 and 18
20. LIMIT 15 to (conference abstract or conference paper or "conference review" or editorial or letter)
21. 15 not (16 or 17 or 19 or 20)
22. Limit 21 to English language

## A. 3. Medline. database

1. Exp infant, low birth weight/ or infant, premature/
2. Exp infant, premature, diseases/
3. Premature Birth/
4. (Preterm or pre-term or prematur*).mp.
5. 1 or 2 or 3 or 4
6. Exp Proteomics/
7. Proteome/
8. Proteom*.tw,kf.
9. 6 or 7 or 8
10. Exp Blood Proteins/
11. (Blood-protein* or serum-protein* or plasma-protein*).tw,kf.
12. Exp biomarkers/
13. (Biomarker* or marker*).tw,kf.
14. 10 or 11 or 12 or 13
15. 5 and 9 and 14
16. (Exp animals/ or (rat or rats or mouse or mice or swine or porcine or murine or sheep or lamb or lambs or pig or pigs or piglet or piglets or rabbit or rabbits or cat or cats or dog or dogs or cattle or bovine or monkey or monkeys or trout or marmoset or marmosets).ti.) not human*.sh.
17. Limit 15 to (case reports or comment or editorial or guideline or letter or practice guideline)
18. 15 not (16 or 17)
19. Limit 18 to English language

## Abbreviations

ROP: Retinopathy of prematurity
PVD: Pulmonary vascular disease
PH: Pulmonary hypertension
LOS: Late onset sepsis
BPD: Bronchopulmonary dysplasia
NEC: Necrotising enterocolitis
GA: Gestational age
Pro-apoC2: Proapolipoprotein CII
SAA: Serum amyloid A
MALDI-TOF MS: Matrix assisted laser desorption ionization-time of flight mass spectrometry
MnSOD: Mitochondrial superoxide dismutase
CRDL1: Chordin-like protein 1
PCSK9: Proprotein convertase subtilisin/kexin type 9
FGF-19: Fibroblast growth factor 19
MSP: Hepatocyte growth factor-like protein
LH: Luteinizing hormone
IGFBP-7: Insulin-like growth factor-binding protein
iTRAQ: Isobaric tags for relative and absolute quantitation
LC–MS/MS: Liquid chromatography and tandem mass spectrometry
C1Inh: C1-inhibitor
SAP: Serum amyloid P
Apo-D: Apolipoprotein D
LRG1: Leucine-rich alpha-2-glycoprotein 1
ZAG: Zinc-alpha-2-glycoprotein
ORM: Orosomucoid
MST1: Macrophage stimulating 1
PF-4: Platelet factor 4
MST1R: Macrophage-stimulating protein
APP: Amyloid precursor protein
STK16: Serine/threonine-protein kinase 16
CTAP-III: Connective tissue-activating peptide III
PDGF-AA: Platelet-derived growth factor AA
VEGF121: Vascular endothelial growth factor 121
ANG-1: Angiopoietin 1
ANG-2: Angiopoietin 2
BMP10: Bone morphogenetic protein 10
HGF: Hepatocyte growth factor
PEA: Proximity extension assays
C3dCR2: Complement C3d Receptor 2
COLEC12: Collectin subfamily member 12
INHBC: Inhibin beta C subunit
SELL: Selectin L
IL2-RA: Interleukin 2 Receptor alpha
GP6: Glycoprotein 6 platelet
GFBP-1: Insulin-like growth factor-binding protein-1
FSTL3: Follistatin like 3
GDF15: Growth differentiation factor 15
CGA: Glycoprotein hormone alpha polypeptide
ELLSA: Enzyme-linked immunosorbent assay
MIF: Macrophage migration inhibitory factor
IUGR: Intrauterine growth restriction
AGA: Adequate gestational age
MBOAT7: Lysophospholipid acyltransferase 7
SUMO3: Small ubiquitin-related modifier 3
FCN2: Ficolin-2
TF: Serotransferrin
PDA: Patent ductus arteriosus
BNP: B-type natriuretic peptide
